# Vitamin D Deficiency Induces Chronic Pain and Microglial Phenotypic Changes in Mice

**DOI:** 10.3390/ijms22073604

**Published:** 2021-03-30

**Authors:** Nicola Alessio, Carmela Belardo, Maria Consiglia Trotta, Salvatore Paino, Serena Boccella, Francesca Gargano, Gorizio Pieretti, Flavia Ricciardi, Ida Marabese, Livio Luongo, Umberto Galderisi, Michele D’Amico, Sabatino Maione, Francesca Guida

**Affiliations:** 1 Department of Experimental Medicine, Università degli Studi della Campania “Luigi Vanvitelli”, 80138 Naples, Italy; nicola.alessio@unicampania.it (N.A.); belardocarmela85@gmail.com (C.B.); mariaconsiglia.trotta2@unicampania.it (M.C.T.); salvatore.paino@unicampania.it (S.P.); boccellaserena@gmail.com (S.B.); flavia.ricciardi@unicampania.it (F.R.); ida.marabese@unicampania.it (I.M.); livio.luongo@gmail.com (L.L.); umberto.galderisi@unicampania.it (U.G.); michele.damico@unicampania.it (M.D.); sabatino.maione@unicampania.it (S.M.); 2 Università Campus Bio-Medico di Roma, 00128 Rome, Italy; f.gargano@unicampus.it; 3 Department of Plastic Surgery, University of Campania “Luigi Vanvitelli”, 80138 Naples, Italy; gorizio.pieretti@unicampania.it

**Keywords:** microglia, vitamin D deficiency, chronic pain, gender, palmitoylethanolamide

## Abstract

The bioactive form of vitamin D, 1,25-dihydroxyvitamin D (1,25D3), exerts immunomodulatory actions resulting in neuroprotective effects potentially useful against neurodegenerative and autoimmune diseases. In fact, vitamin D deficiency status has been correlated with painful manifestations associated with different pathological conditions. In this study, we have investigated the effects of vitamin D deficiency on microglia cells, as they represent the main immune cells responsible for early defense at central nervous system (CNS), including chronic pain states. For this purpose, we have employed a model of low vitamin D intake during gestation to evaluate possible changes in primary microglia cells obtained from postnatal day(P)2-3 pups. Afterwards, pain measurement and microglia morphological analysis in the spinal cord level and in brain regions involved in the integration of pain perception were performed in the parents subjected to vitamin D restriction. In cultured microglia, we detected a reactive—activated and proliferative—phenotype associated with intracellular reactive oxygen species (ROS) generation. Oxidative stress was closely correlated with the extent of DNA damage and increased β-galactosidase (B-gal) activity. Interestingly, the incubation with 25D3 or 1,25D3 or palmitoylethanolamide, an endogenous ligand of peroxisome proliferator-activated-receptor-alpha (PPAR-α), reduced most of these effects. Morphological analysis of ex-vivo microglia obtained from vitamin-D-deficient adult mice revealed an increased number of activated microglia in the spinal cord, while in the brain microglia appeared in a dystrophic phenotype. Remarkably, activated (spinal) or dystrophic (brain) microglia were detected in a prominent manner in females. Our data indicate that vitamin D deficiency produces profound modifications in microglia, suggesting a possible role of these cells in the sensorial dysfunctions associated with hypovitaminosis D.

## 1. Introduction

1,25-dihydroxyvitamin D (1,25D3) is a neuroactive steroid regulating multiple functions in the central nervous system (CNS) [1]. In the brain, 1,25D3 exerts anti-inflammatory and neuroprotective effects through its interaction with vitamin D receptor (VDR), a member of the nuclear receptor family of ligand-activated transcription factors. Upon stimulation, VDR forms a heterodimer with the retinoid X receptor (RXR), and by binding to specific DNA response elements, regulate gene transcription. VDRs are widespread in the brain and spinal cord including the areas involved in the regulation of sensorial behaviors and cognition [1,2]. In the CNS, microglia represent the immune cells responsible for the early defense mechanism following injury or diseases. In pathological situations microglia undergo to activation resulting in the release of several neuromodulators and contribute to the neuroinflammation [3]. Microglia cells express VDR and specific enzyme for converting 25-OH vitamin D (25D3) into its active form 1,25D3 [4,5]. In vitro, the exposure to 1,25D3 reduces the expression of pro-inflammatory cytokines in activated microglia [4]. The inverse correlation between serum 25D3 levels and painful manifestations has been previously reported [6,7]. Remarkably, few studies revealed that vitamin D supplementation decreases pain in patients affected by fibromyalgia or nonspecific musculoskeletal pain [8]. In our previous study, we showed that a vitamin-D-deficient diet causes abnormal pain sensation (allodynia) and spinal neuronal sensitization in male mice, suggesting the role of vitamin D/VDR signaling in the transmission of noxious signals [9]. However, the possible contribution of microglia in the effects induced by Vitamin D deficiency has been not investigated. Thus, given the crucial role of microglia in the pathophysiology of chronic pain, we explored the effects of vitamin D deficiency on microglia cells. For this purpose, we employed a model of low vitamin D intake during gestation to investigate the impact of maternal vitamin D status on brain microglia cells in offspring. Specifically, cultured primary microglia obtained from vitamin D-deficient pups were examined in term of cell viability, proliferation, oxidative stress, and senescence. In vitro microglia were pharmacologically stimulated with 25D3 or 1,25D3, or palmitoylethanolamide (PEA), an endogenous ligand of peroxisome proliferator-activated receptor-alpha (PPAR-α), activating the RXR-mediated signaling pathway. Afterward, the effects of vitamin D deficiency on microglia in parents subjected to Vitamin D-deficient diet were explored. Indeed, pain behavior and microglia morphology in the spinal cord level and in brain regions involved in the integration of pain perception were evaluated. Our data revealed that microglia cells are subjected to dysfunctional modifications in both pups and adult mice in vitamin D deficiency condition.

## 2. Results

### 2.1. Vitamin D Deficiency Promotes Primary Microglia Cells Activation and Proliferation

**Figure 1:**
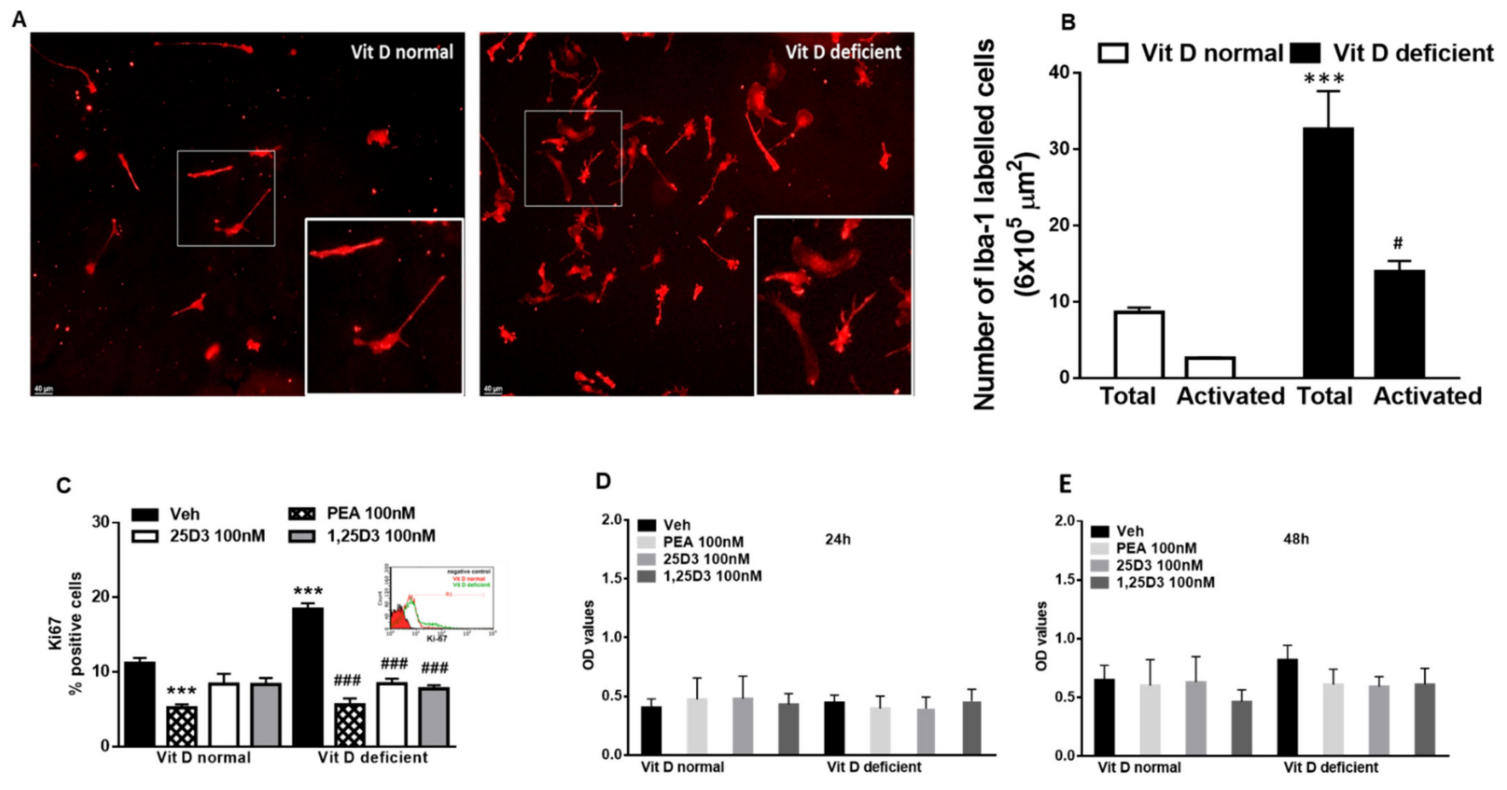
(**A**) Representative staining and related quantification (**B**) of primary microglia cells in Iba-1 labeled microglia cells in normal or deficient Vitamin D (Vit D) conditions. Morphological evaluations measured as the number of total or activated cells. Data are shown as mean ± SEM (cells samples n = 6–8). *** indicates statistically significant values (*p* < 0.0001) vs. total number of VitD-normal cells; # indicates statistically significant values (*p* < 0.05) vs. activated VitD-normal cells. Two-way ANOVA, post-hoc Tukey’s. (**C**) Analysis of Ki67-positive microglia in normal or deficient VitD conditions in the presence of vehicle (DMSO 0.001%, 24 h) or 25D3 or 1,25D3 or PEA (100 nM, 24 h). *** indicates statistically significant values (*p* < 0.0001) vs. vehicle-treated VitD-normal cells; ### indicates statistically significant values (*p* < 0.0001) vs. vehicle-treated Vitamin D-deficient cells (cells samples n = 6–8). (**D**,**E**) Measurement of microglial cell viability (24 h and 48 h) in normal or deficient VitD conditions in the presence of 25D3 or 1,25D3 or PEA (100 nM, 24 h). Data are expressed as Optical density (OD) values (cells samples n = 12). Two-way ANOVA, post-hoc Tukey’s.

Vit D deficiency induces morphological changes in cultured primary microglia. We found an increase of the number of activated cells, as indicated by the enlarged soma diameters and shorter process lengths, with respect to normal Vit D cells that appeared in resting condition (*p* = 0.005) (Figure 1A,B). Interestingly, a substantial increase of cells’ total number was observed in Vit-D-deficient microglia (*p* = 0.0008) (Figure 1A,B). To further investigate cell proliferation, we analyzed Ki67 protein expression by flow cytometric analysis. Compared with the control, Vit D deficiency resulted in a significant (*p* = 0.0002) increase in Ki67-positive cells. This effect was normalized (*p* ≤ 0.0001) by exposing cells to 25D3 or 1,25D3 treatment (100 nM, 24 h). Interestingly, PEA (100 nM, 24 h) reduced Ki67 staining too (*p* ≤ 0.0001), though this effect was also observed in control cells (*p* = 0.004) (Figure 1C). Vitamin D deficiency did not influence the microglia cell cycle (see Supplementary Figure S1).

Control experiments verified that the Vit D deficiency condition, as well as pharmacological treatments (25D3 or 1,25D3 or PEA 100 nM for 24 and 48 h) did not induce significant changes in cells’ viability (Figure 1D,E).

### 2.2. Vitamin D Deficiency Induces ROS Generation in Primary Microglia Cells

**Figure 2:**
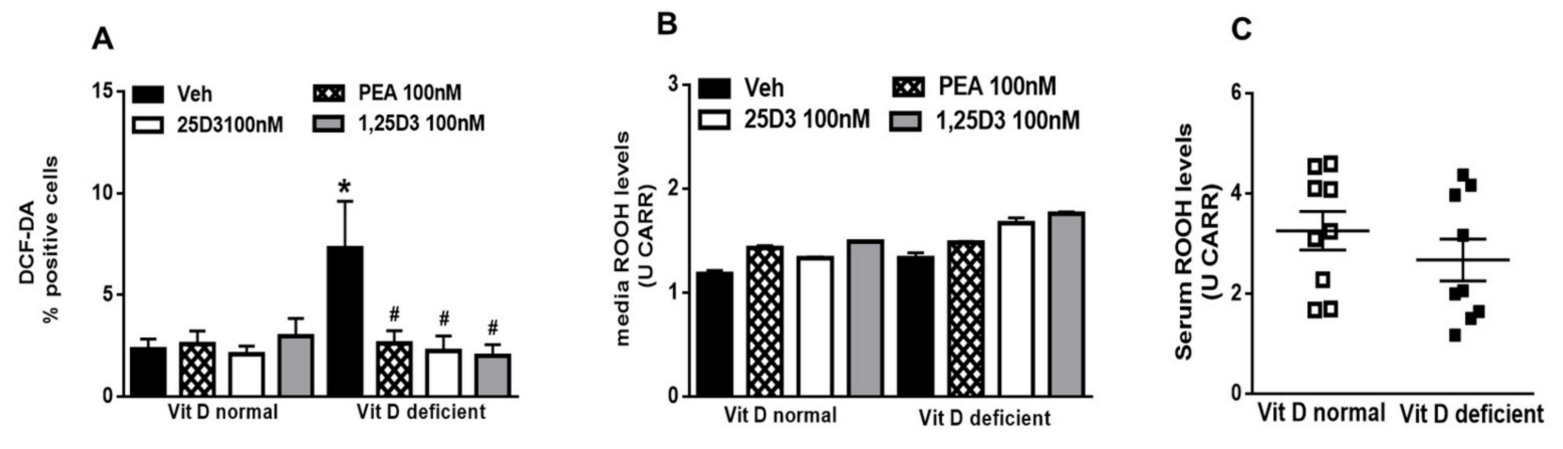
(**A**) Intracellular ROS generation from 2,7-dichlorodihydrofluorescein diacetate (DCFH-DA) assay in normal or deficient Vitamin D (VitD) primary microglia cells. (**B**) Reactive oxygen species (ROS) levels (ROOH production) detected in the cell media of normal or deficient VitD microglia cells. Data were expressed as means ± SEM (U CARR). (**C**) ROS levels (ROOH production) detected in the serum of normal or deficient VitD pups. Data were expressed as means ± SEM (U CARR). Cells were treated were vehicle (DMSO 0.001%, 24 h) or 25D3 or 1,25D3 or PEA (100 nM, 24 h) (cells samples n = 6–8). * indicates statistically significant (*p* < 0.05) difference vs. vehicle-treated VitD-normal cells; # indicates statistically significant difference vs. vehicle-treated VitD-deficient cells. Two-way ANOVA, post-hoc Tukey’s.

To assess the reactive state of the microglia cells, we measured ROS production by DCFH-DA assay. As shown in the Figure 2, Vit D deficiency induced the production of significant (*p* = 0.039) amounts of intracellular ROS in microglia compared with control. ROS levels in the cells incubated with 25D3 (*p* = 0.034) or 1,25D3 (*p* = 0.025), or PEA (*p* = 0.05) (100 nM, 24 h for all treatments) were reduced (Figure 2A). We did not detect any changes in ROS levels in cells media nor in the serum samples obtained from pups (Figure 2B,C, respectively).

### 2.3. Vitamin D Deficiency Increases β-Gal Activity in Primary Microglia Cells

**Figure 3:**
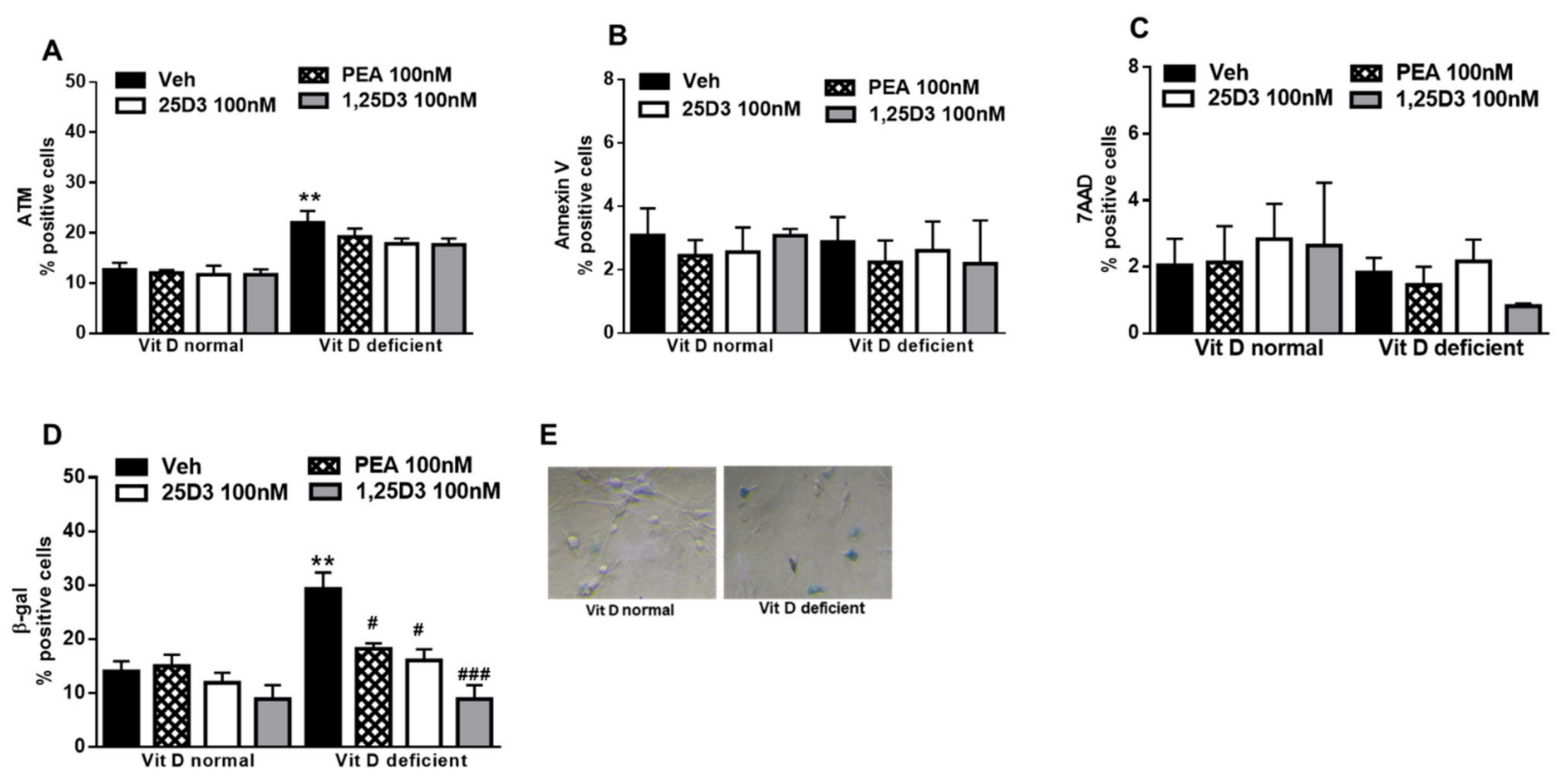
(**A**) Percentage of ataxia-telangiectasia mutated (ATM)-positive normal or deficient VitD primary microglia cells. (**B**) Percentage of Annexin V-positive normal or deficient VitD microglia cells. (**C**) Percentage of 7AAD normal or deficient VitD microglia cells. (**D**,**E**) Percentage of β-Gal in normal or deficient VitD primary microglia cells. Cells were treated with vehicle (DMSO 0.001%, 24 h) or 25D3 or 1,25D3 or PEA (100 nM, 24 h). Values are represented as mean ± SEM (cells samples n = 6–8), ** indicates statistically (*p* ≤ 0.001) significant difference vs. vehicle-treated VitD-normal cells; # and ### indicate statistically (*p* ≤ 0.05 and *p* ≤ 0.0001) significant difference vs. vehicle-treated VitD-deficient cells. Two-way ANOVA, post-hoc Tukey’s.

Acute or chronic intracellular excess of ROS is involved in damage to the DNA, which becomes particularly toxic when encountered by the DNA replication apparatus, necessitating additional mechanisms to deal with such lesions and allow accurate genome duplication. Indeed, in vitamin D deficiency, we found a higher expression (*p* = 0.0044) of ataxia-telangiectasia mutated (ATM), a well-known nuclear protein involved in the initiation of oxidative stress mechanisms and DNA repair signaling. However, the presence of 25D3, or 1,25D3, or PEA was not able to reduce ATM-positive cells numbers, as compared with vehicle (Figure 3A). Cells with extensive DNA damage permanently exit the cell cycle (senescence) or undergo programmed cell death. Here we observed that vitamin D deficiency did not change the apoptosis processes, as revealed by Annexin V detection (Figure 3B). Necrotic cells number was not affected by vitamin D levels (Figure 3C). Differently, the evaluation of β-Gal expression, a marker of cell senescence, revealed that the percentage of positively stained cells markedly increased under vitamin D deficiency (*p* = 0.0018). Interestingly, the appearance of most of the β-Gal-labelling recalled a morphological activated state of the cells. The incubation with 25D3 (*p* = 0.038) or 1,25D3 (*p* = 0.0124) or PEA (*p* = 0.0184) (100 nM, 24 h) reduced the percentage of β-Gal positive cells (Figure 3D,E).

### 2.4. Vitamin D Deficiency Induces Pain Behavior in Adult Male and Female Mice

**Figure 4:**
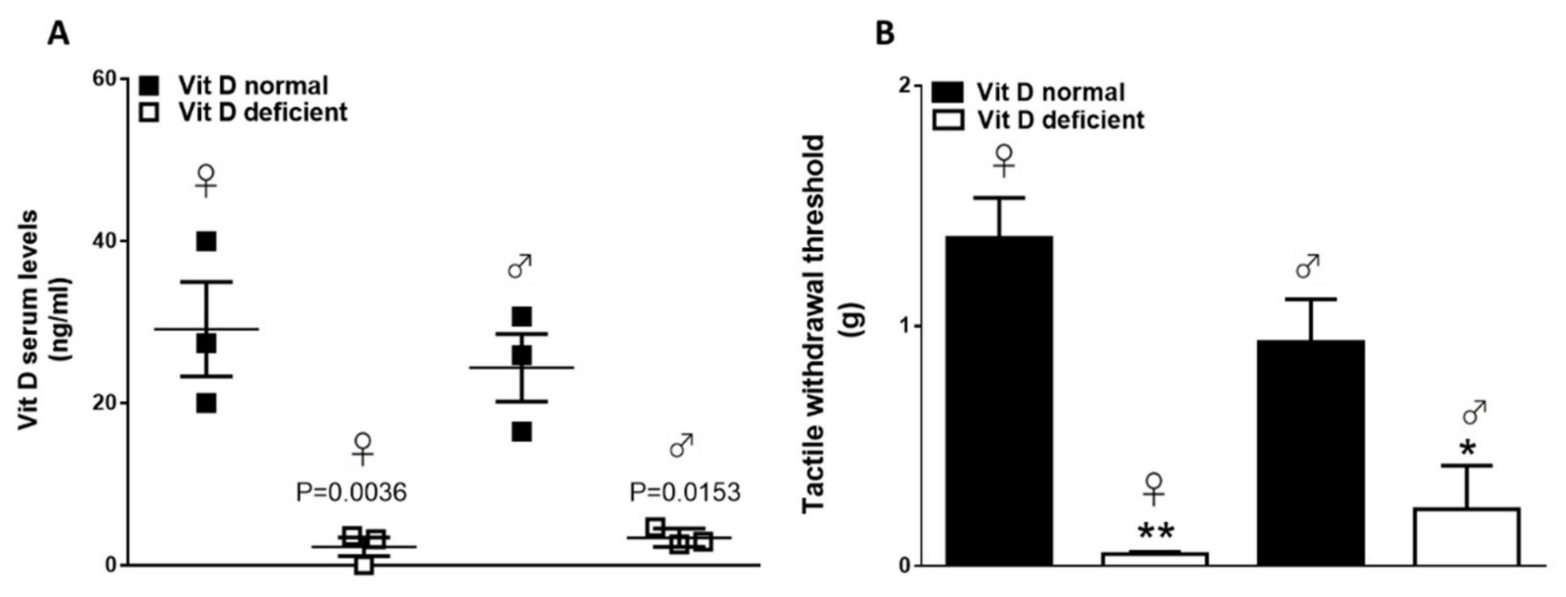
(**A**) Vitamin D serum levels in normal (VitD normal) and vitamin-D-deficient (VitD normal) female or male mice. Data are expressed as mean ± SEM (n = 3 for each group). (**B**) Tactile withdrawal threshold in normal (VitD normal) and vitamin D-deficient (VitD deficient) female or male mice. Data are expressed as mean ± SEM; * and ** indicate statistically significant (*p* ≤ 0.05 and *p* ≤ 0.001) difference vs. VitD normal mice (n = 3 for each group).

Six weeks of the feeding diet containing low vitamin D concentrations resulted in serum 25OHD concentrations significantly lower than those in mice fed with a diet containing normal amounts of vitamin D. 25OHD levels were 29.1 ± 5.8 ng/mL and 24.3 ± 4.1 ng/mL in control mice, while they were 2.2 ± 1.1 and 3.4 ± 0.6 ng/mL, respectively, in females and males in the low-vitamin-D group (Figure 4A; *p* < 0.05). As we previously have shown [9], Vitamin D deficiency induced abnormal pain referred to as a reduced tactile withdrawal threshold at the hind paw plantar surface. In the present study, we also included female mice. When compared with controls, vitamin-D-deficient mice showed reduced tactile threshold in both female and male animals (1.3 ± 0.16 g vs. 0.05 ± 0.01 0.93 ± 0.17 g vs. 0.23 ± 0.18; *p* < 0.05) (Figure 4B).

### 2.5. Vitamin D Deficiency Induces Microglial Morphological Changes in Adult Mice

**Figure 5:**
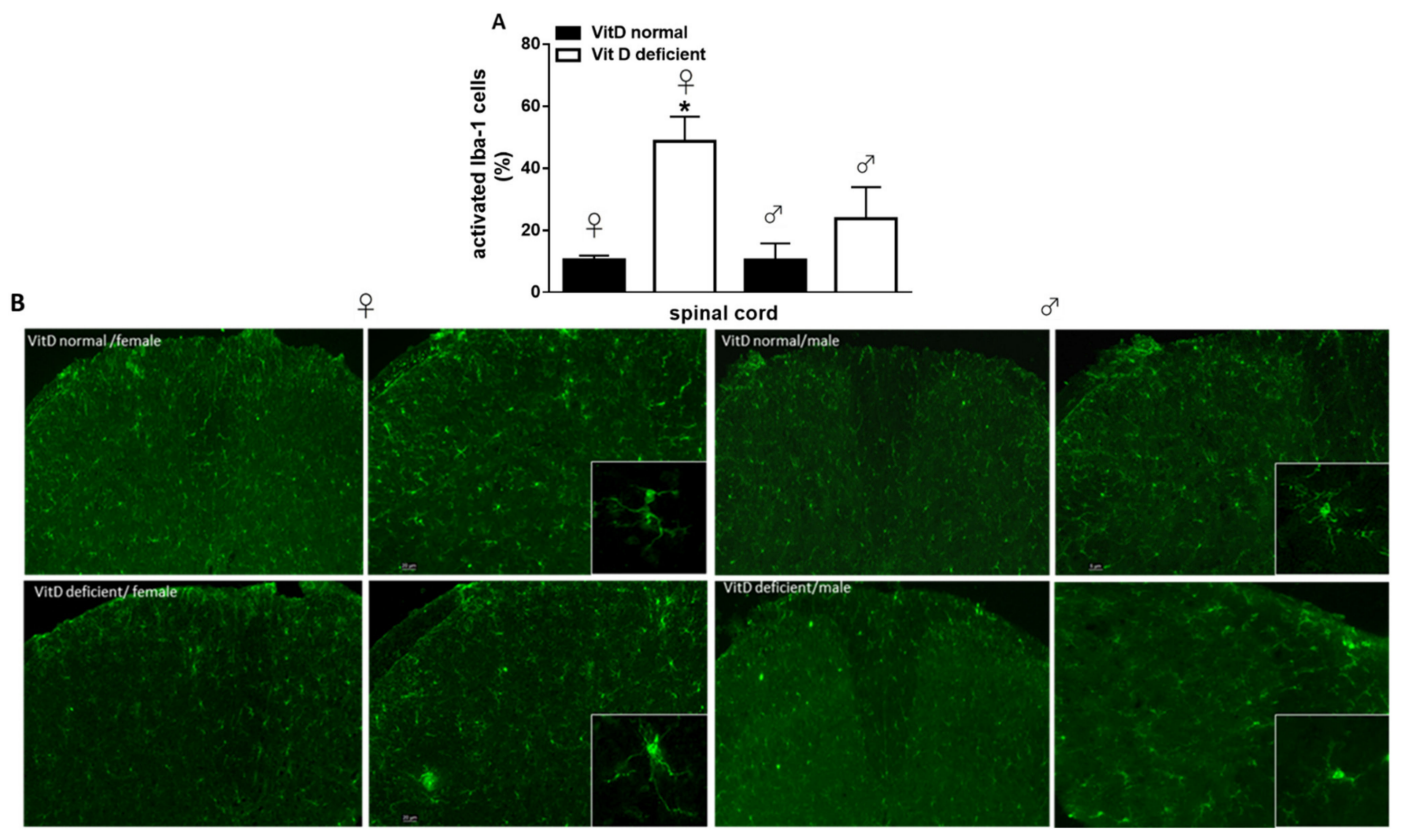
(**A**) Quantitative analysis and (**B**) representative images and of the activated Iba-1 positive cells in the spinal cord of VitD-normal or VitD-deficient female or male mice. Squares represent crop of a single Iba-1 positive profile to highlight the morphology. Data are expressed as the percentage of activated microglia cells vs. the total cells number for each group (n = 3 mice per group). * indicates statistically (*p* ≤ 0.05) significant difference vs. related control. Two-way ANOVA, post hoc Tukey. Scale bars 5 and 20 µm.

**Figure 6:**
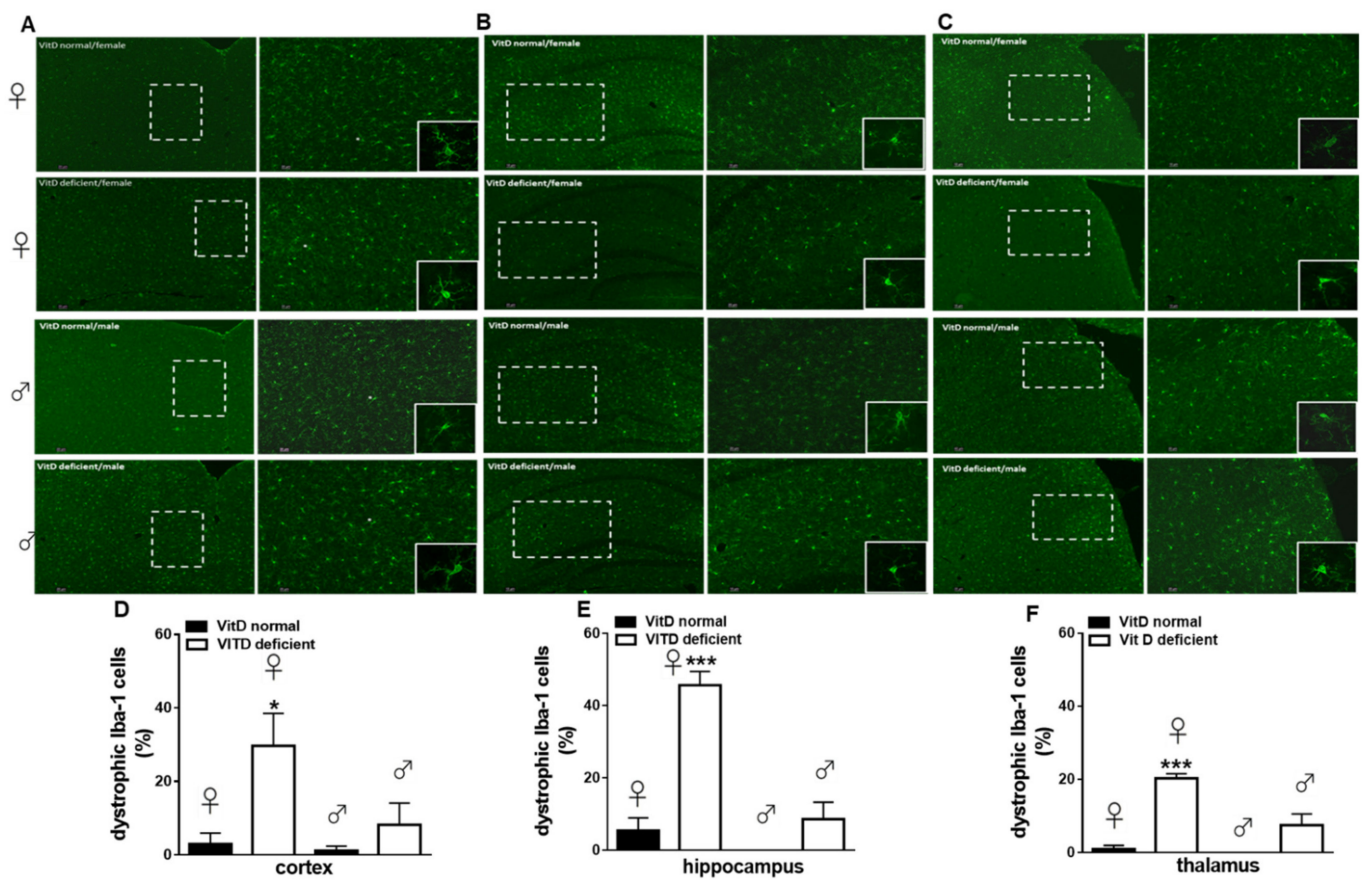
Representative images and quantitative analysis of the dystrophic Iba-1 positive cells in the cortex (**A**,**D**), hippocampus (**B**,**E**), and thalamus (**C**,**F**) of VitD-normal or VitD-deficient female or male mice. Squares represent crop of a single Iba-1 positive profile to highlight the morphology. Data are expressed as the percentage of dystrophic microglia cells vs. the total cells number for each group (n = 3 mice per group). * and *** indicate statistically (*p* ≤ 0.05 and *p* ≤ 0.001) significant difference vs. related control. Two-way ANOVA post hoc Tukey. Scale bars 5 and 20 µm.

Low vitamin D intake caused significant changes of iba-1-positive cells morphology. In the spinal cord, while the total cell number was unchanged (not shown), an increase in the number of activated microglia cells was detected in vitamin-D-deficient animals, as compared with the control (10.43 ± 1.41% of activation in females and 10.36 ± 5.41% of activation in males). Interestingly, this effect was prominent in females (48.71 ± 7.91% of activation, *p* = 0.020), while in male mice, it was not significant (23.7 ± 10.21% of activation; *p* = 0.1321) (Figure 5A,B). Two-way ANOVA analysis showed significant differences for treatment (diet regimen) (F1,8 = 13.44, *p* = 0.0063), but not significant differences for sex (F1,8 = 3.17, *p* = 0.112). Moreover, no treatment x sex interaction was found (F1,8 = 3.13, *p* = 0.114). Low vitamin D diet did not significantly change the total number microglia in all brain regions analyzed, as compared with the control diet (not shown). No significant changes in the number of activated cells were found. However, an increase in the number of dystrophic microglia was observed at the cortical level in female (29.73 ± 8.77%, *p* = 0.036), but not in male mice (8.23 ± 5.87%, *p* = 0.80), as compared to Vit D-normal mice (3 ± 3% for females and 1.23 ± 1.23 for males) (Figure 6A,D). Two-way ANOVA analysis showed significant differences for treatment (F1,8 = 9.32, *p* = 0.015) but not significant differences for sex (F1,8 = 4.43, *p* = 0.068). Moreover, no treatment x sex interaction was found (F1,8 = 3.19, *p* = 0.111). Likewise, the number of dystrophic microglia dramatically increased in the hippocampus (45.66 ± 3.84% of total cells, *p* = 0.0002) and thalamus (20.29 ± 1.31% of total cells, *p* = 0.0002) of Vit-D-deficient females, compared to Vit D-normal mice (5.51 ± 3.51% and 1 ± 1%), respectively (Figure 6B,C,E,F). Any change in the dystrophic cells number was detected in neither the hippocampus (8.66 ± 4.66% of total cells, *p* = 0.359), nor in the hypothalamus (7.53 ± 3.03% of total cells, *p* = 0.059) of male mice compared with Vit D-normal mice (0 ± 0% of total cells) (Figure 6E,F). Indeed, increase in % of dystrophic cells in hippocampus (*p* = 0.0003) and in the thalamus (*p* = 0.0035) resulted greater in Vit-D-deficient female mice than male mice (Figure 6E,F). Two-way ANOVA analysis showed significant differences for treatment (F1,8 = 48.75, *p* = 0.0001) for sex (F1,8 = 36.97, *p* = 0.0003), and a treatment x sex interaction was found (F1,8 = 20.28, *p* = 0.020), for the hippocampus. Moreover, Two-way ANOVA analysis showed significant differences for treatment (F1,8 = 60.35, *p* < 0.0001) and for sex (F1,8 = 15.88, *p* = 0.0040), and a treatment x sex interaction was found (F1,8 = 11.60, *p* = 0.0093), for the thalamus.

## 3. Discussion

Low vitamin D levels are common in people suffering from chronic pain, but the mechanism through which vitamin D status affects pain modulation is unclear. Our previous study demonstrated that low vitamin D intake produces pain behavior and spinal neuronal overexcitability, together with an increase of the spinal VDR expression levels, suggesting a maladaptive plasticity possibly due to changes in vitamin D-related signaling. Based on the central role of microglia in the onset and maintenance of chronic pain, we aimed to investigate the effects of vitamin D deficiency on microglia cells. To assess this, we used a dual approach by characterizing microglia cells in both pups and adult mice subjected to a low vitamin D intake.

Cultured microglia obtained from vitamin-D-deficient pups appeared with cytoskeletal rearrangements that are typical of morphological cell activation, as characterized by a larger somata and shorter processes than control cells. Moreover, the total cell number was increased, and the Ki67 overexpression proved that in vitro microglial cells proliferate in response to the vitamin D deficiency. VDR activation has been demonstrated, in highly cell-specific manners, to alter cellular proliferation through multiple mechanisms, most prominently via effects on cell cycle progression, apoptosis, and differentiation [10]. The antiproliferative and anti-apoptotic effects of 1,25D have been reported in different experimental conditions [11]. Therefore, the increased cell proliferation observed here might result from the altered genomic VDR signaling caused by Vitamin D absence. Indeed, vitamin D addition in cell media reduced this effect, as indicated by the normalized Ki67 expression. Interestingly, 25D and 1,25D induced superimposable effects, supporting the finding that microglia cells, as neurons, own the enzymes responsible for the Vitamin D active form synthesis [5]. As sensors of the surrounding environment, microglia are highly responsive to local perturbations. It is accepted that, beside the typical switch between a resting state and an activated (pro-inflammatory) state, stimulated microglia can exhibit a range of phenotypes in response to damage-associated stimuli. A few studies suggested that early life exposure (pre- and post-natal) to stress conditions, including infections and diet regimens, can interfere with microglial developmental programs, leading to long-lasting alterations, which could be implicated in later behavioral abnormalities [12]. Here we found that Vitamin D deficiency induces reactive—activated and proliferative—microglia accompanied by an increased ROS generation. High levels of ROS likely contribute to microglial oxidative stress associated with chronic pain and neurodegeneration, while appropriate ROS levels have been shown to be indispensable for cell survival, apoptosis, and differentiation [13]. In our system, we found a 3-fold-increase of intracellular ROS production, while cell apoptosis or necrosis was not changed. It could be possible that vitamin D deficiency may be sufficient to “prime” microglia cells to be more responsive to subsequent challenges. We cannot exclude that the augmented proliferation of microglia (ki67 overexpression) could be the consequence of the increased oxidative stress associated with the low vitamin D condition in cell cultures. On the other hand, vitamin D deficiency may cause a progressive loss of cell functioning. Indeed, a previous study by Djukic and colleagues [14] reported that Vitamin D deficiency reduces the E. coli phagocytosis in microglia, suggesting an impaired resistance against bacterial infections. Interestingly, vitamin D absence induced increased expression of ATM in microglia, indicating an enhanced DNA damage repair efficiency. Our data are consistent with previous studies showing the reduced DNA damage signaling by 1,25D3 in lymphocytes, which would also be coherent with the antioxidant properties of this hormone [15].

Moreover, we detected an increase of β-Gal expression, a commonly used biomarker of cell senescence. Although senescence would be apparently in contrast with the proliferative activity of Vitamin D-deficient microglia, we cannot exclude the possibility of a partial in vitro selection process that allows only non-senescent cells to proliferate. On the other hand, as others have previously suggested, SA-β-gal activity cannot be attributed uniquely to cell senescence, but it may be considered a ubiquitous marker of cellular stress [16]. In our previous study, we showed that the administration of PEA, the endogenous ligand of PPAR-a-RXR-mediated signaling pathway, was able to reduce pain behavior in vitamin D-deficient mice [9]. The anti-inflammatory and neuroprotective effects of PEA have been previously demonstrated in other pathological conditions [17,18,19,20]. Here we found that PEA exerted protective effects on microglia cells similarly to 1,25D, suggesting a common downstream pathway. In fact, the interaction between VDR and PPAR-α, both requiring heterodimerization with RXR to exert their activity through response element binding, has been previously suggested [21]. Based on our findings, one can speculate that PEA could reduce vitamin D deficiency-induced pain by targeting microglia cells, but further experiments are necessary.

Immunohistochemistry was used to investigate possible modifications of microglia in adult mice subjected to the low vitamin D intake. Interestingly, the proliferative activity of microglia observed in vitro in vitamin D deficiency was not detected in the spinal cord or brain of adult mice. This discrepancy between in vitro and ex vivo results could be in part due to the different experimental conditions, such as the age and sex of the animal used. However, we revealed morphological changes of microglia in the spinal cord and in different brain regions. Before that, we observed that the allodynia previously reported in male mice under Vitamin D deficiency [9] was also present in females. In these latter animals, spinal microglia appeared hypertrophic, as suggested by the swollen soma, and retracted processes. These data suggest the participation of spinal microglia, possibly in a pro-inflammatory phenotype (M1), in allodynia associated with Vitamin D deficiency. We have previously reported that increased spinal VDR expression could be responsible for modifications in VDR-mediated pathway with subsequent modulation of transduction signals participating in pain processes, including endocannabinoid system [9]. Because microglia express cannabinoid receptors in addition to being capable of synthesizing and degrading their endogenous ligands, we cannot exclude that endocannabinoid system may regulate microglia phenotype in Vitamin D-deficient animals. However, the analysis of microglial M1/M2 polarization will be needed to clarify the microglia phenotype in our model.

At supraspinal levels, microglia participate to the neuroinflammation, which constitute the “common soil” of the multifactorial diseases, including neurodegenerative disorders. Under chronic pain, brain microglial cytokines overproduction has believed to play a major role in the affective/cognitive comorbidities, including depression and memory deficit [22]. In our study we show that vitamin D deficiency causes profound changes in brain regions involved in the integration of sensorial perception (pain), emotional stimuli, and cognition. In the cortex, thalamus, and hippocampus, we observed an increased number of dystrophic microglia. Microglial dystrophy represents the morphological manifestation of senescence. Dystrophic rather than activated microglia have been associated with tau pathology and likely precede neurodegeneration in Alzheimer’s disease in humans [22,23,24]. On these bases, we can speculate that microglia changes in vitamin D deficiency may be predictive of a brain malaise that could, in turn, mirror in a malfunction of the whole-brain neural network.

Remarkably, activated (spinal) or dystrophic (brain) microglia were detected in a prominent manner in females. This finding seems to be in accordance with previous studies suggesting that vitamin D-mediated beneficial effects may be female-specific, also based on the evidence that estrogens promote the potency of vitamin D neuroprotective properties [25]. Moreover, although males and females may display similar behavioral dysfunctions, the gender-dependent immune-driven induction of chronic pain has been suggested [26].

Based on our data, we can speculate that microglia might not participate in the peripheral sensitization in vitamin D deficiency in males at least at this time point, suggesting the participation of alternative immune-neural interactions.

Given the presence of high levels of VDR in the brain [27], and also based on the fact that 1,25D3 doses used in our study may reflect the range of concentrations to which microglia would likely be exposed, it is reasonable that vitamin D deficiency may directly affect microglia functioning.

On the other hand, few evidence suggested that lifestyle (i.e., high-fat diet or physical exercise) may change brain microglia morphology, while functional consequences remain to be elucidated [28,29]. Thus, we cannot exclude that metabolic alterations in lipid metabolism or in gut microbiota metabolites, commonly associated with vitamin D deficiency, could indirectly affect microglia physiology.

## 4. Materials and Methods

### 4.1. Animals and Experimental Design

**Scheme 1:**
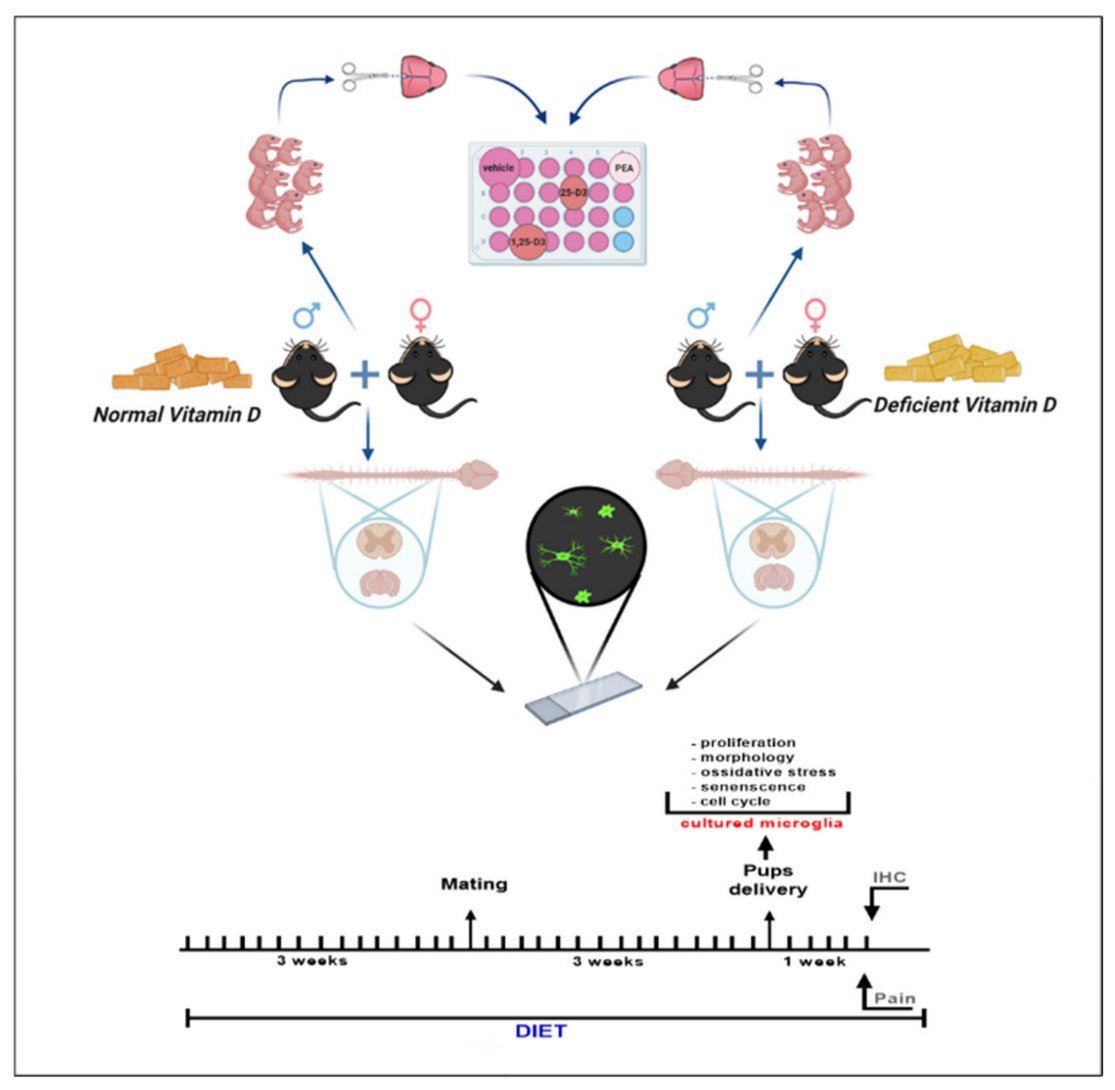
Scheme showing experimental design of the study. The image was created with BioRender drawing software.

Male and female C57Bl/6J mice (3 weeks) obtained from Envigo Laboratory were housed controlled illumination and environmental conditions. The experimental procedures were approved by the Animal Ethics Committee of University of Campania of Naples. Animal care was in compliance with the IASP and European Community (E.C. L358/1 18/12/86) guidelines on the use and protection of animals in experimental research. All efforts were made to minimize animal suffering and to reduce the number of animals used. Mice were fed a diet with low vitamin D concentration (<5 IU/kg cholecalciferol, purified vitamin D3 deficient diet, Art No E15312-24) or with normal vitamin D concentration (1500 IU/kg cholecalciferol, purified control diet, Art No E15000-04) for three weeks before timed mating, and diet exposure was maintained until the end of the experiment. Mice were randomly assigned to the diverse diet regimens. In compliance with the principles of the 3Rs (Replacement, Reduction, and Refinement), we employed the minimum number of animals previously reported for similar experiments. Both diets were identical in terms of all other components including calcium and phosphorus and were obtained from Ssniff, Soest, Germany [30]. Pups (P2-4) mice, whose parents were fed a vitamin-D-deficient or vitamin-D-normal diet, were used for microglia cell culture protocol. One week after delivery, adult mice were submitted to the behavioral testing and then sacrificed for microglia morphological analysis. The experimental design is given in Scheme 1.

### 4.2. Primary Microglial Cultures

Primary microglia were prepared according to our previous studies [9,31]. A total number of 45 pups for each condition (normal or deficient vitamin D diet) was used. Briefly, the brains of C57/Bl6 postnatal (P2-4) mice were dissociated and suspended in Dulbecco’s modified Eagle medium (DMEM). The cell suspension was filtered through a 100-µm nylon mesh (BD Biosciences, UK) and plated (in tissue culture flasks precoated with poly-D-lysine (10 µg/mL). Cells were maintained in a 5% CO_2_ incubator at 37 °C and were harvested as floating cell suspensions following shaking after 10–12 days. Cells were plated in multiwell plates and after 24 h were treated as follows: Vehicle (DMSO 0.001%, 24 h), or 25-dihydroxyvitamin D3 (25D3) (100 nM, 24 h), or 1,25-dihydroxyvitamin D3 (1,25D3) (100 nM, 24 h), or palmitoylethanolamide (PEA) (100 nM, 24 h). The dose used was chosen from a pilot experiments preceding this study. Cellular samples were obtained by 12–15 pups and each set of experiment was performed three times (three different dams/broods). Each sample was evaluated in duplicate (technical replicate). Evaluations were performed by a blind observer. 25-dihydroxyvitamin D3 or 1,25-dihydroxyvitamin D3 were purchased from Tocris, Bristol, UK. Ultramicronized palmitoylethanolamide was provided by Epitech Group SpA (Saccolongo, Padova, Italy).

### 4.3. Immunocytochemistry

For this set of experiments, cells were plated on coverslips and then treated as follows: Vehicle, or 25D3, or 1,25D3 or PEA. After 24 h, they were fixed in 4% paraformaldehyde for 20 min, followed by ice-cold 100% methanol for 3 min. Fixed cells were incubated for 2 h with primary antibodies against Iba-1 (rabbit anti-ionized calcium binding adapter molecule 1; 1:1000 dilution; Wako Chemicals, Germany). Following this incubation, cells were washed and incubated for 45 min with the secondary antibody solution. Coverslips were mounted using Vectashield mounting media (Vector Laboratories, Burlingame, CA). Activated cells were detected by measuring the cell soma and processes length. Resting and activated microglia were classified based on the following criteria: Resting microglia displayed small somata bearing long, thin, ramified processes, whereas activated microglia exhibited marked cellular hypertrophy and retraction of processes such that the process length was lower than the diameter of the soma compartment [8]. For detection of Ki67 or ataxia-telangiectasia mutated (ATM), cells were collected and fixed in 4% formaldehyde (Sigma-Aldrich, MO, USA) solution from 15′ at RT. Cells were treated with 0.3% of Triton-X100 solution (Roche, Switzerland) in ice for 5 min, followed by blocking solution (5% FBS solution in PBS and 0.1% Triton-X100) for 1h a RT. Subsequently, the cells were incubated in a blocking solution with the antibodies Ki67 (sc-7846, Santa Cruz, TX, USA), or ATM (ab36810, ABCAM, UK) for O.N. at 4°C. Subsequently, cells were washed three times with PBS, before adding the secondary antibody FITC conjugate were obtained from ImmunoReagents (NC, USA). Samples were acquired on a Guava EasyCyte flow cytometer (Merck Millipore MA, USA) and analyzed with a standard procedure using EasyCyte software.

### 4.4. Measurement of Primary Microglial Cells Proliferation

A Cell Counting Kit-8 (CCK-8) (Dojindo Molecular Technologies, Germany) was used for the determination of cell viability. The amount of the formazan dye, generated by the activities of dehydrogenases in cells, was considered directly proportional to the number of living cells. 24 h and 48 h cell viability was detected following the manufacturer’s instructions. The assay was performed by a microplate reader at 450 nm (Infinite 2000, TECAN, Switzerland). The data were expressed as OD values at 24 h and 48 h.

### 4.5. Cell Cycle Analysis of Primary Microglial Cultures

Cells were collected and fixed in 70% ethanol at −20 °C, O.N., followed by PBS 1X washes, and finally were dissolved in a hypotonic buffer containing propidium iodide (Sigma-Aldrich MO, USA). Samples were acquired on a Guava EasyCyte flow cytometer (Merck Millipore MA, USA) and analyzed with a standard procedure using EasyCyte software.

### 4.6. Nexin V Assay in Primary Microglial Cultures

Apoptotic and necrotic cells were detected using a fluorescein-conjugated with Nexin V kit on a Guava EasyCyte (Millipore, Italy) flow cytometer, following the manufacturer’s instructions. The kit used two separate dyes (annexin V and 7AAD) to identify apoptotic and necrotic cells. Annexin V (green) binds to phosphatidylserine on the external membrane of apoptotic cells, while 7AAD (red) permeates and stains DNA of late-stage apoptotic and dead cells. Staining allows the identification of 3 cell populations: Necrotic cells (annexin V- and 7AAD+); early apoptotic cells (annexin V+ and 7AAD-); late-apoptotic or dead cells (annexin V+ and 7AAD+). In our experimental conditions, early and late apoptotic cells were grouped together.

### 4.7. DCF-DA Assay in Primary Microglial Cultures

The generation of reactive oxygen species (ROS) was monitored by the conversion of fluorogenic 2,7-dichlorodihydrofluorescein diacetate (DCFH-DA) to highly fluorescent dichlorofluorescein diacetate within cells by ROS. The cells were incubated for 30 min at RT with 2 μM DCFH-DA in PBS and 0.1% Pluronic F-127 (Sigma-Aldrich, MO, USA). Cells were washed with PBS acquired on a Guava^®^ easyCyte™ flow cytometer (Millipore, Sigma) and analyzed using easyCyte™ software.

### 4.8. D-ROM Test

Serum samples obtained from pups or collected media obtained from cells were collected. From both studies, 5μL of samples were used to measure D-ROM (Diacron, Grosseto, Italy) following the manufacturer’s instructions, which was adapted to an autoanalyzer (Infinite m200, TECAN, Switzerland).

### 4.9. In Situ Senescence-Associated Beta-Galactosidase Assay in Primary Microglial Cultures

Cells were fixed using 2% formaldehyde and 0.2% glutaraldehyde. After that, cells were washed with PBS (Microgem, Italy) and then incubated at 37 °C with staining solution pH 6 (40 mM citric acid/Na phosphate buffer, 5 mM K4[Fe(CN)6] 3H2O, 5 mM K3[Fe(CN)6], 150 mM sodium chloride, 2 mM magnesium chloride and 1 mg ml − 1 X-gal in distilled water). The percentage of was calculated by the number of blue, b-galactosidase-positive cells out of at least 300 cells in different microscope fields, as already reported [32]. All reagents where not differently specify were obtained from Sigma-Aldrich (MO, USA).

### 4.10. Serum Vitamin D Measurements

Serum samples of adult mice were centrifuged and kept frozen at −20 °C until analysis. The levels of [25(OH)D] (ng/mL) were measured by quantitative chemiluminescent microparticle immunoassay (CMIA) Architect 25-OH Vitamin D (Abbott Laboratories).

### 4.11. Behavior in Adult Mice

Mechanical allodynia was assessed by using a Simplified Up-Down method (SUDO), consisting of a modified version of the up-down method introduced by [33]. Male and females adult mice were placed on a wire mesh platform and, 1 h after habituation period, calibrated von Frey filaments (North Coast Medical, Gilroy, CA, USA; ranging from 0.002 to 2.0 g bending force) were applied to the hind paw for 3 to 4 s. Five von Frey filament presentations per test were executed and a lack of response to a filament dictates that the next highest filament is used in the following stimulation, while a positive response dictates the use of the next lowest filament. Data were showed as tactile withdrawal threshold (grams).

### 4.12. Ex Vivo Immunohistochemistry in Adult Mice

Under pentobarbital anesthesia (50 mg/kg, i.p.), male and female animals were transcardially perfused with saline solution followed by 4% paraformaldehyde in 0.1 M phosphate buffer. The brains and spinal cords were excised, post fixed for 3 h in the perfusion fixative, cryoprotected for 72 h in 30% sucrose in 0.1 M phosphate buffer, and frozen in Optimal cutting temperature-embedding compound. Dorsal horn of spinal cord, prelimbic/infralimbic cortex, hippocampus, and ventral posterolateral nucleus of thalamus were analyzed. Transverse sections (20 µm) were cut using a cryostat and thaw-mounted onto glass slides. Slides were incubated overnight with primary antibody solutions for the microglial cell marker Iba-1 (rabbit antiionized calcium binding adapter molecule-1; 1:1000; Wako Chemicals, Germany). Possible non-specific labeling of rabbit secondary antibody was detected by using secondary antibody alone. Following incubation, sections were washed and incubated for 2 h with secondary antibody solution (donkey anti-rabbit Alexa FluorTM 488; 1:1000; Molecular Probes, USA). Slides were washed, coverslipped with Vectashield mounting medium (Vector Laboratories, USA), and visualized under a Leica fluorescence microscope. Quantitative analysis was performed by counting in areas measuring 1.7 × 104 µm^2^. Nine sections for each animal were analyzed. The experiments have been performed by a blind observer. According to our previous studies [28], Iba-1-positive cells were identified as resting (with small somata bearing long, thin, and ramified processes), activated microglia (with hypertrophy together with retraction of processes to a length shorter than the diameter of the somata), or dystrophic microglia. Dystrophic microglia were recognized by debris consisting of several cells displaying fragmented processes and an irregularly shaped cell body as previously demonstrated [34].

## 5. Statistical Analysis

Cells’ biomolecular data and behavior were represented as mean ± S.E.M. analyzed using Two-way ANOVA, followed by post-hoc Tukey’s. Immunohistochemical data were expressed as percentage of relative control and were analyzed by Two-way ANOVA, post-hoc Tukey’s.

## 6. Conclusions

In conclusion, our data indicate that vitamin D deficiency produces profound modifications in microglia, suggesting that their dysfunction may contribute to sensorial and neurological alterations often associated with hypovitaminosis D status.

## Supplementary Materials

The following are available online at https://www.mdpi.com/article/10.3390/ijms22073604/s1.
